# Optimization of training and measurement protocol for eNose analysis of urine headspace aimed at prostate cancer diagnosis

**DOI:** 10.1038/s41598-021-00033-y

**Published:** 2021-10-22

**Authors:** Laura Capelli, Carmen Bax, Fabio Grizzi, Gianluigi Taverna

**Affiliations:** 1 Department of Chemistry, Materials and Chemical Engineering “Giulio Natta”, Politecnico Di Milano, piazza Leonardo da Vinci 32, 20133 Milan, Italy; 2 Department of Immunology and Inflammation, IRCCS Humanitas Research Hospital, via Manzoni 56, Rozzano, 20089 Milan, Italy; 3 Humanitas University, Via Rita Levi Montalcini 4, Pieve Emanuele, 20090 Milan, Italy; 4 Department of Urology, Humanitas Mater Domini Hospital, Via Gerenzano, 2, 21053 Castellanza, Varese Italy; 5 Department of Urology, IRCCS Humanitas Research Hospital, via Manzoni 56, Rozzano, 20089 Milan, Italy

**Keywords:** Cancer screening, Prostate cancer, Diagnostic markers

## Abstract

More than one million new cases of prostate cancer (PCa) were reported worldwide in 2020, and a significant increase of PCa incidence up to 2040 is estimated. Despite potential treatability in early stages, PCa diagnosis is challenging because of late symptoms’ onset and limits of current screening procedures. It has been now accepted that cell transformation leads to release of volatile organic compounds in biologic fluids, including urine. Thus, several studies proposed the possibility to develop new diagnostic tools based on urine analysis. Among these, electronic noses (eNoses) represent one of the most promising devices, because of their potential to provide a non-invasive diagnosis. Here we describe the approach aimed at defining the experimental protocol for eNose application for PCa diagnosis. Our research investigates effects of sample preparation and analysis on eNose responses and repeatability. The dependence of eNose diagnostic performance on urine portion analysed, techniques involved for extracting urine volatiles and conditioning temperature were analysed. 192 subjects (132 PCa patients and 60 controls) were involved. The developed experimental protocol has resulted in accuracy, sensitivity and specificity of 83% (CI_95%_ 77–89), 82% (CI_95%_ 73–88) and 87% (CI_95%_ 75–94), respectively. Our findings define eNoses as valuable diagnostic tool allowing rapid and non-invasive PCa diagnosis.

## Introduction

Prostate cancer (PCa) represents the fifth most frequent cancer in the world. Based on GLOBOCAN 2020, more than one million new cases of prostate cancer were reported worldwide in 2020, with higher prevalence in developed countries^1^. A trend towards an increase of PCa incidence worldwide (i.e., + 79.7% overall change) up to 2040 is estimated. Despite its long latency period and potential treatability in early stages, PCa diagnosis is challenging because of late onset of symptoms and limits of current screening procedures based on prostate specific antigen (PSA) blood testing. Although PSA based screening has been associated with a significant reduction in PCa mortality, it has also resulted in over-diagnosis and overtreatment of indolent PCa, exposing many men to treatments without benefits^2,3^. Its low specificity is mainly attributable to the fact that serum PSA values may increase (i.e., > 4 ng mL^−1^) in benign conditions, such as benign prostatic hyperplasia and chronic prostatitis^3^. Additionally, serum PSA levels are affected by biologic variability related to differences in androgen levels, prostate manipulation or racial and ethnic differences^4,5^. Therefore, the currently most widely diagnostic method in men with increased PSA values is the biopsy sampling. However, this procedure is invasive, entails a low level of accuracy (i.e., only 30% detection rate at first biopsy) and is prone to various complications, including sepsis and death^6,7^. There is, thus, an urgent need for more reliable and non-invasive methods to detect PCa at early stage, and differentiate different tumour grades. Recent advances in carcinogenesis proved that cell transformation leads to peroxidation of membrane components and consequent release in biological fluids of volatile organic compounds (VOCs), such as TMPRSS2^8,9^ or PCA3^10^ or metabolites involved in various pathways associated with cells demand and production of energy (e.g., 6-dimethyl-7-octen-2-ol, Dihydroedulan IA, 3,5-dimethylbenzaldehyde and 2-ethylhexanol whose decrease in prostate cancer samples and Formaldehyde, Pentanal, 2,5-Dimethylbenzaldehyde, 3-methylphenol (m-Cresol) or Phenol)^11,12^, which can be used as indicators of cancer presence^12–16^. Evidences underlined the fundamental role of urine, among other biological fluids, as a source of information for diagnostic, prognostic, and predictive purposes, especially for urological diseases^17^. Urine has the advantages of being inexpensive, rich in metabolites, easy to handle, and available in large amounts, without requiring invasive treatments for collection^18^. Even though many studies, focusing on chemical characterization of urine samples to identify specific PCa biomarkers^19^, have been reported in the scientific literature^20–26^, the critical investigation of relevant literature pointed out that this research field requires continue advances. Results achieved until now are fragmented, partial and, in some cases, contradictory: a high number of metabolites has been proposed as suitable PCa biomarkers, although divergent opinions upon the same metabolites is emerged^13,27^. It seems that PCa development is more likely associated to the alteration of the concentration of a pool of compounds without a specific trend, thereby suggesting looking at urine as a whole, instead of focusing on the concentration trend of single metabolites. An alternative approach, proposing the analysis of urine as a whole, entails the analysis of odours emanated from urine samples. Already in 400 BC, Hippocrates recognized the diagnostic usefulness of body odours, reporting different disease-specific odours emanated from urine, skin, and other fluids^28^. The most promising results reported in the literature were obtained relying on trained dogs’ olfaction for detecting various cancers^29–34^. Canine olfaction can perceive odour thresholds as low as parts per trillion. Thus, dogs can trace the presence of a unique odour signature, despite the complexity of body fluids due to their extremely variable and diluted composition^13^. In 2015, Taverna et al.^35^ proved the capability of two trained dogs to detect PCa by simply smelling urine with an accuracy above 97%. Their results proved the existence of a specific urine odour pattern associated to PCa presence. Nevertheless, this approach is hardly implementable in the clinical practice because its lack of reproducibility and scalability.

In 2016, taking advantage from a multi-disciplinary collaboration between the Humanitas Mater Domini Hospital in Castellanza, Varese (Italy) and the Politecnico di Milano University (Italy), we decided to develop an Electronic Nose (eNose)^36^ to reproduce dogs’ capability to discriminate urine samples from PCa patients and controls to implement an effective and potentially large-scale diagnostic tool. eNoses have been already studied in the biomedical field for discriminating bacteria cultures or detecting urinary tract infections, diabetes or kidney diseases through the analysis of biological fluids^37–42^, and some preliminary feasibility studies concerning early PCa diagnosis have also been published^43–46^. Most of them characterized volatiles emanated from urine samples (i.e., headspaces) aiming to identify a PCa-specific odour fingerprint. However, experimental protocols involved for sample preparation and eNose analysis differed considerably. Very different conditioning temperature were proposed to enrich urine headspace and various approaches (e.g., dynamic or static headspaces) were involved for eNose analysis^13^. Despite a significant number of papers regarding the use of eNoses for urine analysis, the scientific literature lacks specific studies investigating the influence of choices concerning the experimental protocol on eNose discriminative capability between urine from controls and men affected by PCa. Given the variability associated with urine samples^37^, those aspects can significantly affect results’ reproducibility and influence the classification performance^13^. Consequently, up to now, for researchers approaching the field of urine analysis by eNoses for diagnostic purposes, there are no clear indications about suitable and most effective experimental procedures to be adopted to maximize the instruments’ discrimination capability. With the aim to provide an advancement of knowledge in this field, the research here presented focuses on the definition of an optimized experimental protocol for urine analysis by deeply investigating the dependence of eNose responses on different aspects concerning sample preparation and analysis. Here we describe the approach aimed at improving the experimental protocol for eNose application for PCa diagnosis. The effects of the portion of urine analysed, the techniques involved for enriching the urine headspace and the conditioning temperature on instrument classification capability were investigated.

## Results

### Dependence of eNose response on the portion of urine analysed

**Figure 1:**
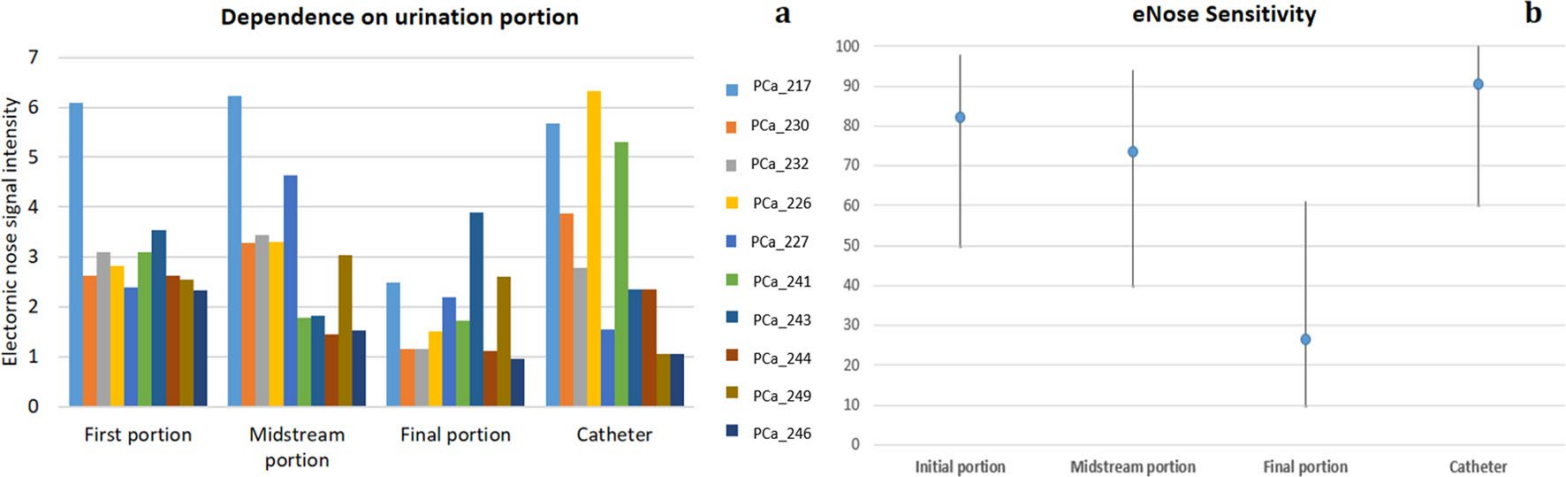
Comparison of eNose (**a**) responses and (**b**) sensitivity relevant to the analyses of different portions of urine samples from the same subjects of the Prostate cancer group (PCa).

Three urine samples relevant to first, midstream and final portions of urination and a catheter sample were collected from PCa patients and analysed by eNose, to investigate the potential influence of the portion of urination on the eNose signals and, as a consequence, on the eNose capability to detect PCa presence. The comparison of eNose responses relevant to the analyses of different portions of urination and catheter sample from the same subjects highlighted that the intensity of eNose signals relevant to the analysis of final portion of urination was significantly lower than ones recorded for first and midstream portions (Fig. 1a). The intensity of sensor signals was measured as the ratio between the resistance value at the beginning of the analysis and the minimum value reached when the sensor was exposed to urine headspace. This evidence could be explained by the anatomy of male urethra and the washing effect occurring in first part of urination. In males, urethra connects urinary bladder to urinary meatus for the removal of urine from the body, crossing through the prostate gland^47^. Thus, the concentration of metabolites from the prostate is at maximum levels in first portion of urination and decreases progressively during urination^48^. Conversely, the analysis of catheter sample, representing a mix of different portions, is comparable to the analysis of initial portions, but its collection is invasive. Thus, the analysis of spontaneously collected sample is preferred.

Due to the poorer concentration of metabolites from the prostate, the analysis of the last portion did not provide sufficient information for discriminating PCa samples from controls. Indeed, a sensitivity of only 27% (CI_95%_ 10–61) compared to 81% (CI_95%_ 50–98), 75% (CI_95%_ 40–94) and 91% (CI_95%_ 60–100) was achieved respectively for the first, midstream portion of urination, and catheter sample (Fig. 1b). These results further support a first evidence reported by Asimakopoulos et al.^46^, who pointed out a positive correlation between a positive prostate biopsy and the analysis by a sensor array of first portion of urination, and a poor correlation of the clinical status of patients with signals relevant to the analysis of the last portion of urination. Based on results achieved, the standardized procedure for collecting samples from PCa patients and controls involved the distinction of different urination portions and only the first portion was analysed for identifying PCa.

### Dependence of eNose response on the enrichment temperature

**Figure 2:**
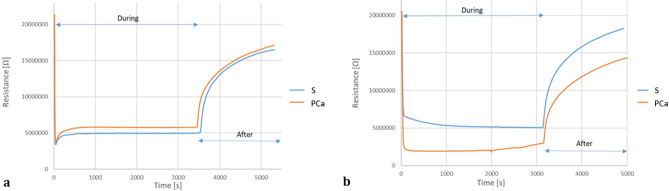
Example of eNose signals relevant to the analysis of urine headspaces from PCa patient and controls enriched at (**a**) 37 °C and (**b**) 60 °C.

Different tests carried out under various experimental conditions to define the optimal protocol for sample preparation pointed out a strict dependence of eNose signals on the temperature at which the liquid urine was conditioned to enrich the gaseous phase. Indeed, the increase of the conditioning temperature resulted in a significant increase of eNose signal amplitude relevant to urine headspaces from PCa patients rather than controls, thereby resulting in a better classification performance. This outcome, most likely related to the increase of metabolites (mainly volatile organic compounds) concentration in urine headspaces enriched at higher temperatures^49^, underlined the need of including in the sample preparation procedure for eNose analysis a conditioning step at higher temperature to enhance eNose detection capability, differing from what proposed by Taverna et al.^35^ with trained dogs. Artificial olfaction is not as powerful as canine olfaction in detecting odour down to part per trillion. eNose lower detection limit towards most odorants of interest is in the order of tens of ppb^50,51^. Thus, conditioning temperatures of 50 °C and 60 °C were tested. Although there was the possibility of compounds in the urine reacting if the enrichment temperature was increased, the strict sampling regime adopted ensured that all urine samples were exposed to the same conditions during headspace creation and so any side reactions had the same chance of occurring for all samples^52^. A maximum temperature of 60 °C to prevent protein denaturation and samples degradation during the enrichment was adopted^53^. Figure 2 reports eNose signals relevant to the analysis of urine headspaces from PCa patient and controls enriched at 37 °C and 60 °C, respectively. For clarity, we decided to show only the resistance ratio of the sensors most solicited by urine headspaces, which plays a crucial role in the detection of PCa volatiles (i.e., zinc oxide-based sensor). This aspect has been the aim of our recent study published in the Journal of the Electrochemical Society^54^.

Because of enhanced sensors’ responses, eNose classification performance of samples enriched at higher temperatures is improved. eNose signals recorded for urine headspaces enriched at 23 °C and 37 °C resulted in many false negatives, since they did not provide enough information to distinguish among urine from controls and PCa patients. Instead, eNose classification performance based on the analyses of samples enriched at 50 °C and 60 °C proved a promising capability of the innovative tool to discriminate among urine samples from controls and PCa patients, with an accuracy close to 80%. Table 1 shows the results achieved for the same urine samples enriched at 50 °C and 60 °C, acquired for a subset of the 178 subjects involved in the study, were compared. With the same specificity, eNose sensitivity is significantly improved for headspaces conditioned at 60 °C (i.e., 78%(CI_95%_ 74–83%)) than ones enriched at 50 °C (i.e., 69%(CI_95%_ 64–74%)). Based on results achieved, the proposed experimental protocol foresees the enrichment of static headspaces at 60 °C.

**Table 1:** Comparison of eNose diagnostic performance based on the analysis of urine headspaces enriched at 50 °C and 60 °C.

Test characteristics S vs PCa	Conditioning temperature (°C)	
	50	60
Accuracy (CI_95%_)	74% (69–79%)	79% (74–84%)
Sensitivity (CI_95%_)	69% (64–74%)	78% (74–83%)
Specificity (CI_95%_)	84% (79–89%)	83% (78–88%)

### Repeatability of eNose responses among different subjects

**Figure 3:**
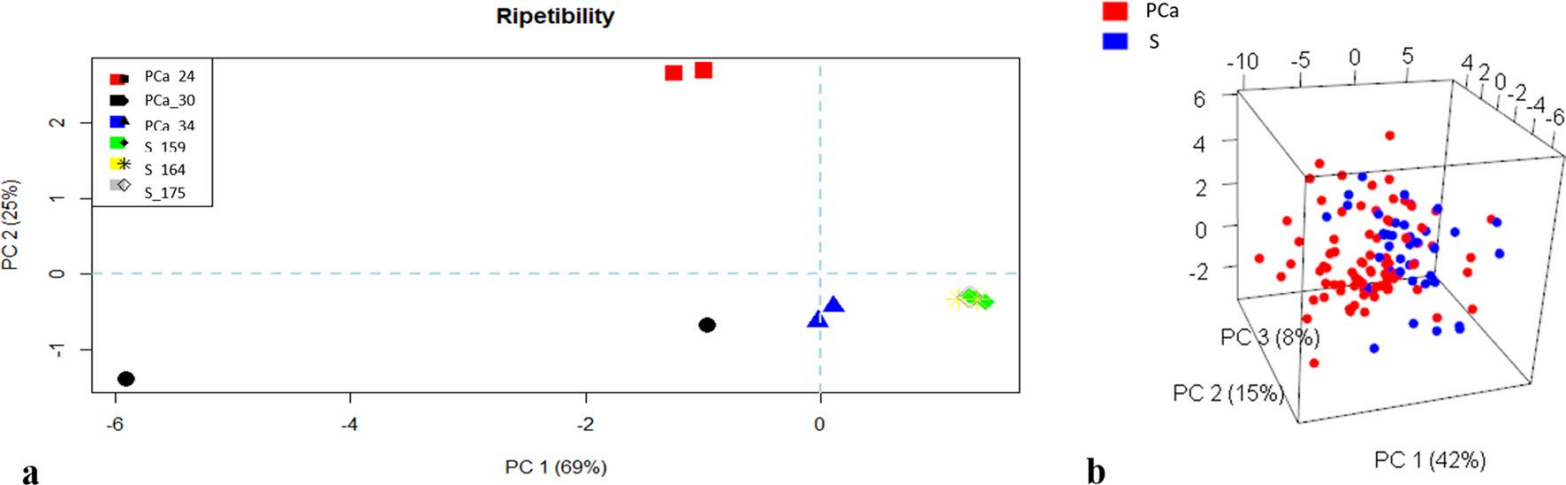
(**a**) Repeatability of e-nose responses relevant to urine samples from the same subjects; (**b**) PCA score plot relevant to the PCa diagnosis model (*S* control, *PCa* prostate cancer group).

Given the variability of urine samples not only among subjects, but also among different samples from the same subject, the repeatability of the proposed experimental protocol was evaluated. For a subset of the population involved in the study, different urine samples (first portion of the urination) were collected on different days and analysed by eNose, according to the defined experimental procedure. This investigation aimed to study the influence of diet, physical activity or any other factor that may have affected urine composition on eNose capability to correctly recognized samples from the same subject and discriminate PCa patients from control. No exclusion criteria, concerning dietary habits, smoking, taking medications were applied. Moreover, no strict rules about the hour of the collection was fixed. Figure 3a reports the Principal Component Analysis (PCA) score plot relevant for those analyses, which enables a visual inspection of multidimensional datasets. The score plot, reporting the projection of samples into a new coordinate system, defined by principal components (i.e., a linear combination of signals provided by different sensors), provides information about data similarities and can be used to investigate the existence of trends or grouping among samples. Samples from the same subject cluster in the same area of the plot, proving that the proposed experimental protocol is robust, and that results are not affected by the intrinsic variability of urine samples. Indeed, the distance among points in a PCA score plot is inversely proportional to their similarities^55^. This result confirmed that no exclusion criteria are needed to ensure good classification performances, as it was suggested by Taverna et al.^35^.

### Prostate cancer diagnosis

Data relevant to the analysis of urine headspaces enriched at 60 °C were organized in a dataset, consisting of 164 samples (110 PCa patients and 54 controls), and the data processing procedure, described in Materials and Methods, was applied to build the diagnosis model. Among the 150 features considered, the feature selection by Boruta identified 37 features (transient and steady-state) as relevant for the discrimination of samples from controls and PCa patients, which were used to build the classifier by randomForest. Figure 3b reports the PCA score plot relevant to PCa diagnosis model. Samples belonging to different class clustered in different region of the plot, highlighting the potentialities of eNose to distinguish PCa samples from controls. Most of the control samples (blue circle) distributed in the right portion of the plot, even though some outliers were present, while the PCa samples (red circle) distributed mainly in the left region of the plot. The innovative diagnostic tool based on urine odour analysis by eNose and the above-described analysis protocol achieved an accuracy of 83% (CI_95%_ 77–89), with a sensitivity of 82% (CI_95%_ 73–88) and a specificity of 87% (CI_95%_ 75–94). The results of the 10-fold cross validation are summarized in Table 2.

**Table 2:** Classification results from 10-fold cross validation on urine samples from PCa and control groups.

	Clinical condition		Test characteristics	% (CI_95%_)
	*S*	*PCa*		
**eNose classification**			Accuracy	83 (77–89)
*S*	47	20	Sensitivity	82 (73–88)
*PCa*	7	90	Specificity	87 (75–94)

## Discussion

Innovative approaches based on urine odour analysis that have been proposed as an alternative to the current PCa diagnostic procedure can be grouped as (a) sensorial and (b) instrumental methods^13^. Both of them are aimed at identifying cancer-specific “odour fingerprints” through the characterization of urine olfactory properties as a whole. Sensorial methods directly rely on animal sense of smell, while senso-instrumental methods are based on instruments that mimic the mammalian olfaction (i.e., eNoses)^13^. Among sensorial approaches, which proved the capability of trained dogs to distinguish between samples from PCa patients and controls by achieving very promising results in terms of diagnostic sensitivity and specificity^29–35^, the findings obtained by Taverna et al.^35^ has inspired the present research. Their study was carried out according to a sound and rigorous experimental design. Taverna et al.^35^ defined a procedure for urine sample collection and preparation and for dogs’ training, aiming to limit the potential interferences of external factors on dogs’ discriminative capability. Their study involved a large series, i.e., 902 participants (362 PCa patients and 540 controls). Control group included healthy non-pregnant women and healthy men between 18 and 25-year-old with a family negative history for PCa, representing the most distant condition from PCa; men older than 45-year-old with a family negative history for PCa, negative Digital Rectal Examination (DRE) and serum PSA level < 1 ng mL^−1^ or serum PSA level < 2.5 ng mL^−1^ that had been stable with time who had urological and/or systemic disease, and men with serum PSA level < 2.5 ng mL^−1^ that had been stable with time who had urinary obstruction treated with Trans-Urethral Resection of the Prostate (TURP) for Benign Prostatic Hypertrophy (BPH) were included. In their study, two 3-year-old female German Shepherd Explosion Detection Dogs were trained to recognize urine samples from PCa patients. Both dogs achieved an accuracy over 97%, which is considerably higher than the performance of current diagnostic tools based on PSA serum level and prostate biopsy (i.e., 58%^56^). Despite the great performance and the advantages of the canine method as potential diagnostic tool due to its non-invasiveness, simplicity and rapidity of analysis, testing and interpretation of results^51^, trained dogs are not suitable for a large-scale application for different reasons:

- Dogs require an adequate training that is costly and highly time-consuming to develop the discriminative ability.
- The breed involved and the specific methodology used to teach the animals (e.g., type of training and blind test, site, frequency and duration of training, number of runs) may influence the performance.
- Dogs would not be able to work for a few hours consecutively.
- The canine method does not comply with hospital protocols for biological sample analysis.

Given these limitations, some researchers started investigating the possibility to transfer those experimental observations to an instrumental method based on eNose analysis of urine samples^43–46,57,58^, and results achieved proved the eNose capability to distinguish between samples collected from men suffering from PCa and controls with very promising diagnostic accuracy (i.e., close to 90%)^13^. Although most of the literary works proposing the eNoses adoption for PCa diagnosis focus on urine headspace characterization, the critical investigation of the relevant literature pointed out that no uniform experimental protocol has been proposed. Different techniques for enriching the urine headspace and various conditioning temperatures have been proposed, thereby resulting in contrasting evidences. Bernabei et al.^57^ proposed the creation of a steady headspace, by injecting 10 mL of urine in a 2L sterile bag pre-filled with nitrogen at 25 °C. Urine headspace was analysed by an eNose equipped with quartz crystal microbalances sensors, but no information about the duration of eNose analysis was reported. Alternatively, Roine et al.^43^ proposed the creation of a urine headspace by pipetting defrosted urine to a plate heated and maintained at 37 °C. The urine headspace was fluxed into the sensor chamber for 15 min. Then, reference air was applied to restore sensors’ baseline. Conversely, D’Amico et al.^44^, Asimakopoulos et al.^46^ and Santonico et al.^58^ created a dynamic urine headspace by putting urine in a sterile box equipped with dedicated top for continuous injection of odourless air, to strip urine volatiles and enrich the headspace. Nevertheless, no information about the conditioning temperature was provided. In this case, the urine headspace was analysed by an eNose for about 15 min. In 2016, Aggio et al.^45^ proposed the combination of a GC with a MOS sensor to analyse a static urine headspace. According to proposed experimental protocol, urine sample was defrosted in water bath at 60 °C for 30 s, mixed with 0.75 mL of 1 M sodium hydroxide to control the pH and re-immersed in water bath at 60 °C for 50 min. Then, the headspace was injected into the GC-MOS sensor system for the analysis, which lasted about 42 min.

The lack of information about the influence of different choices concerning experimental protocol definition on eNose prediction capability and the complexity associated to urine samples boosted the research here presented, whose main goal is to provide useful information to researchers approaching this field of urine odour characterization by means of eNoses for diagnostic purposes. Given the variability of urine samples not only among different subjects, but also among various samples from the same subject due to many external factors (e.g., diet, physical activity, taking medicines)^37,59^, the research focused on the optimization of an experimental protocol for urine odour analysis by eNose. The influence of choices concerning specifically sample preparation and analysis on the eNose discriminative capability were deeply investigated. The present research follows the promising results reported in 2015 by Taverna et al.^35^, who proved the existence of a unique odour pattern of urine samples from men suffering from PCa. According to what suggested by Taverna et al.^35^, eNose training was designed according to the principle of progressive complication of the system: the population included men suffering from non-metastatic prostate cancer (i.e., PCa group) and baby premature girls, young women, and healthy men between 20 and 60 years old (i.e., Control group). The inclusion of female participants and the gradual introduction of young and older men in the Control group allowed evaluating eNose responses to a progressively more complicate system that approached gradually the condition of men suffering from PCa. To define an experimental protocol capable to provide stable and reproducible results, many tests were carried out under different experimental conditions for sample collection and preparation (e.g., distinction of urination portions, dynamic or static sampling, conditioning temperature and storage time). The comparison of eNose signals relevant to the analysis of samples representative of different portion of urination highlighted a significant decrease of sensitivity for the analysis of the last portion of urination, probably related to the progressive decrease of prostatic metabolites concentration in urine occurring during urination. Thus, the standardized sample collection protocol foresees the distinction of different urination portions, and the analysis of the only first portion for identifying prostate cancer. Concerning sample preparation procedure, the results of various tests, involving different conditioning temperatures (i.e., 23, 37, 50 and 60 °C), pointed out a strict dependence of eNose signals on temperature at which liquid urine was conditioned to enrich the headspace, which significantly influenced the eNose classification capability. eNose signals recorded for headspaces enriched at 23 °C and 37 °C resulted in many false negatives, since they did not provide enough information to distinguish among urine from controls and PCa patients. Conversely, eNose classification performance based on the analyses of samples enriched at 60 °C achieved an accuracy above 80%. Based on these results, the proposed experimental protocol consists of five phases: (a) urine sample collection and storage at − 18 °C; (b) thawing; (c) urine headspace enrichment at 60 °C for 1 h; (d) static headspace extraction and modulation of moisture content; and (e) eNose analysis.

**Figure 4:**
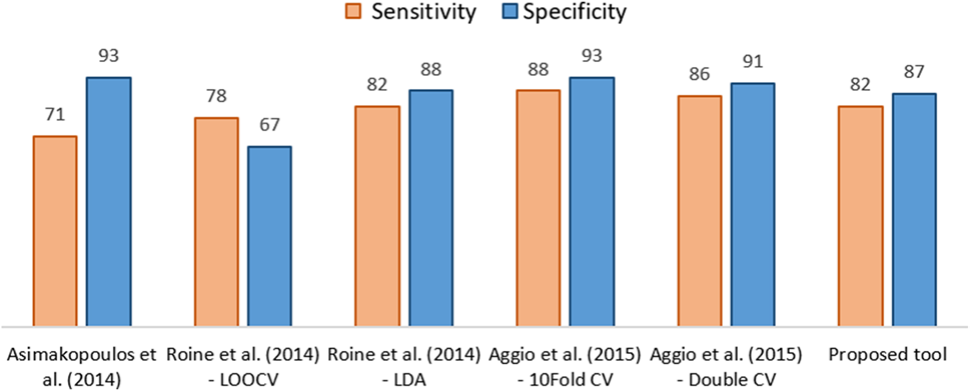
Comparison of the classification performance of the proposed diagnostic tool with ones achieved by different literary studies.

As a result of the optimization of the experimental protocol, which allowed to obtain stable and reproducible sensors’ signals, the classification performance achieved in this research project appears particularly encouraging and shows the opportunity of developing a non-invasive and reliable diagnostic tool for the early PCa detection based on urine odour analysis. Thanks to the high specificity achieved (i.e., 87% (95% CI 75–94%)), the eNose might provide in the future a solution to one of the main issues related to the current diagnostic procedure, i.e. patient’s overtreatment, and the associated high health spending due to high false-positive rates^60^. The classification performance achieved and the population involved were compared to the ones reported in other studies regarding the use of eNoses for PCa detection by means of urine odour analysis^43–46,58^ (Fig. 4). The results achieved within the project here described proved a diagnostic capability of the innovative tool comparable to the one reported in other studies using eNose for the detection of PCa. Compared to the results reported by Asimakopoulos et al.^46^ and Roine et al.^43^, the results achieved within the project here described can be considered to be more robust, since they are based on a significantly larger population: the present project has involved up to now 192 subjects compared to 41 considered by Asimakopoulos et al.^46^ and 74 involved by Roine et al.^43^. Compared to the research by Aggio et al.^45^, who involved a population closer to the one considered within the present study and achieved a comparable diagnostic performance, the instrument here proposed is cheaper and simpler than the hybrid tool combining gas chromatography (GC) with a MOS sensor (GC-MOS). Thus, it might be more easily switched to a large-scale screening tool. In fact, a fundamental aspect for developing a method that might become of widespread use in clinical diagnosis is the switch from a complex laboratory apparatus to an easy-to-use instrument. The use of chemical analysers, as it is the case of the GC, requires highly specialised personnel, whereas the field of clinical diagnosis is rapidly evolving towards point-of-care tests, which ideally should be used by non-specifically trained staff^13^. The critical investigation and the comparison of different approaches proposed in the scientific literature for data processing highlighted that the adoption of different techniques for assessing the performance of classifiers, e.g., LOOCV (leave-one-out cross validation), 10-fold CV (tenfold cross validation), LDA (linear discriminant analysis), or Double CV (double cross validation) affects the sensibility and the specificity achieved. Actually, results reported by Roine et al.^43^ differed significantly, if LOOCV or LDA technique was adopted, even if the same data were considered. Conversely, Aggio et al.^45^, involving more robust data processing procedure, obtained comparable validation results for 10-fold CV and double CV. This points out that the optimization of data processing methods is a crucial aspect of this research field, which should be further investigated in the future.

Despite our important findings, the present research has some limitations and suggest future studies. To confirm the performance of innovative tools and speculate about their use in the clinical practice, a large-scale study, involving a rigorous validation by means of specific blind tests, needs to be carried out. Another critical aspect that needs to be addressed before eNoses may become large-scale diagnostic tool, is the long-term stability of the decisional model. Drift (i.e., the deviation of eNose responses over time under the same stimulus) has limited up to now eNoses industrial applications^61^, because of the progressive decrease of the classification performance under sensor exploitation. To do this, the possibility to develop specific drift correction models for urine headspace datasets, capable to monitor sensor responses and modify the positivity criteria of the decisional model adopted for sample classification based on instrument performance, needs to be investigated. Another aspect, which future works should face with is the transfer ability of prediction models. The method implemented on one instrument should be transferable to other devices without requiring a new training phase to match the scalability requirement of screening tools. Moreover, the specificity of the proposed method toward PCa should be assessed by including in the population patients suffering from other diseases that might interfere with PCa diagnosis.

## Materials and methods

### Electronic nose

The eNose involved for the study is a lab-scale prototype developed at the Politecnico di Milano, equipped with 6 Metal Oxide Sensors (MOS) produced at the Department of Chemistry, Materials and Chemical Engineering of the Politecnico di Milano by inkjet printing^62,63^. The interaction of MOS sensors with volatile organic compounds (VOC) of urine samples by adsorption results in a variation of the sensors’ electrical resistance. During exercise, the MOS sensors were maintained at a constant temperature of about 400 °C by the 5 V powered heater, and the resistance of the metal oxides active layers was acquired continuously by means of a custom-made circuitry at a frequency of 1 Hz and recorded for further processing^54^.

During operation, odourless air is continuously pulled by a vacuum pump through a tube at a flowrate of 50 mL min^−1^ into the chemically inert box, where the sensor array is housed, to create a baseline for the sensor response.

During the analysis, the sample-handling unit exposes the sensors to the odour sample, producing a response that reaches a steady state condition in a few minutes. During this interval (i.e., response time of the sensor array), the sensors response is recorded and delivered to the signal-processing unit. Then, the reference air is again applied to the sensor array to restore the reference line and prepare the sensors for a new measurement cycle.

### Population

The study involved 192 subjects: 132 men suffering from PCa and 60 healthy subjects, recruited at the Humanitas Mater Domini Hospital in Castellanza, Varese (Italy), between 2016 and 2018. Each participant was informed about the study and provided directly or from a parent and/or legal guardian informed consent as participants’ urine was used for the PCa VOC test. The study was approved by the ethical committee at Humanitas Clinical and Research Center, where patients were treated (Approval no. CE-ICH260/11). Patients and the public were not involved in the study design, data collection, analysis, or interpretation of data. All study methods were carried out based on the Declaration of Helsinki.

Subjects were divided in two groups: (a) Prostate cancer Group and (b) Control Group. The PCa Group included: (a) 118 men affected by PCa of different grade and stage treated with open or robotic radical prostatectomy, (b) 7 men with histological diagnosis of PCa at the biopsy and (c) 7 men affected by metastatic PCa (4 subjects; age: 71; 66–84 years) o receiving hormonal therapy (3 subjects; age: 69; 61–79 years) for biochemical relapse after radical prostatectomy or radiotherapy.

The Control Group included urines collected from healthy, non-pregnant, younger and older female volunteers, healthy younger and older male volunteers with a family history negative for PCa, and negative DRE.

Tables 3 and 4 report the baseline characteristics and clinical features of PCa Group and Control Group, respectively.

**Table 3:** Baseline characteristics of prostate cancer group.

Prostate cancer group						
	N (132)	Age (years) Mean (range)	PSA serum level (ng/mL) Mean (range)	Clinical stage	Pathological Gleason Score	Pathological stage
Low risk PCa	23	61 (52–74)	7.26 (3–17.5)	T1c	3 + 3	pT2a–pT2c
Intermediate risk PCa	47	65 (50–78)	7.32 (1.5–29)	T1c	3 + 4	pT2a–pT3
High risk PCa	48	63 (50–77)	10.8 (0.60–62)	T1c–T3	> 3 + 4	pT2a–pT3b
PCa at prostate biopsy	7	59 (46–75)	5.4 (3.2–9.0)	T1c	3 + 3; 3 + 4; 4 + 3	
PCa biochemical relapse	3	69 (61–79)	1 (0.6–2.1)			
Metastatic PCa	4	71 (66–84)	12 (10–78)

**Table 4:** Baseline characteristics of control group.

Control group			
	N (60)	Age (years) Mean (range)	PSA serum level (ng/mL) Mean (range)
Female	19	34 (10–60)	–
Male	35	45 (18–82)	< 2.50
	6	60 (50–75)	3.00 (2.00–5.50)

In line with what suggested by Taverna et al.^35^, the eNose training procedure was defined according to the principle of progressive complication. Urine samples from female subjects were firstly compared with ones from PCa patients. The choice of healthy female participants was dictated by the need to be certain of the absence of specific prostate VOCs in the Control group in the first part of the research, to evaluate the capability of the eNose to distinguish between two certainly different conditions, i.e., men suffering from PCa vs healthy girls. Even though women do not have the prostate, the hormonal changes related to menses might alter eNose responses, thereby women samples might interfere with eNose classification of samples from the PCa group. Thus, baby premature girls younger than 12 years old, representing the most distant condition from prostate cancer were first considered. Then, samples from young women between 20 and 35 years old were gradually introduced to study the effects of hormonal variability on the discrimination between cancerous and healthy urine samples. Further, the complexity of the system was progressively increased by considering young men, subjects with very low probability of contracting PCa (i.e., < 5%), and finally older men, increasing the probability of undetected prostate cancer incidence^35^. Indeed, that probability is lower than 5% for men younger than 27 years old, lower than 10% for men younger than 35 years old and higher than 50% for men over 60 years old^35^. All men included in the Healthy group were considered healthy based on the outcomes of actual diagnostic protocol for PCA detection. Some young men who underwent surgical treatments for correction of phimosis and varicocele and men suffering from Benign Prostatic Hypertrophy (BPH) were also included in the Control group to evaluate the possible interference of these prostatic diseases with cancer detection.

Female urines were used only for preliminary evaluations of the eNose capability to differentiate different classes, and the reproducibility of the experimental protocol in case of interfering factors, while the classification model here presented included as Controls only male subjects.

### Urine samples

For each subject, 4 urine samples, differentiating among first, intermediate and final portion of urination and catheter sample, were collected in sterile urine containers. Common urine analysis that ensures the absence of infectious diseases were performed on urine samples before the storage. Men affected by prostate cancer provided urine samples before prostate biopsy, Transurethral Resection of the Prostate (TURP) or radical prostatectomy, while control samples were collected after routine clinical exams.

#### Sample preparation and analysis

The definition of an optimized experimental protocol for sample preparation and analysis by eNose represented the main aspect of this research, since it can significantly affect the eNose classification performance. This is particularly true in the case of complex odour matrixes, as it is the case for urine, which is constituted by thousands of metabolites and characterized by intrinsic variability due to lots of factors (e.g., diet, physical activity, etc.)^37,59,64^. Given the high number of degrees of freedom involved in urine sample preparation and analysis, the problem was tackled considering each step separately: urine sample storage, sample preparation, eNose analysis. The following subsections describe the different steps of the experimental procedure.

#### Urine sample storage

After collection, urine samples are stored at − 20 °C to inhibit bacterial cultures eventually present and prevent modifications of the composition of the samples just produced. Frozen samples are transported under controlled temperature to the Department of Chemistry, Materials and Chemical Engineering “Giulio Natta” of the Politecnico di Milano and stored until use at − 18 °C in different compartments according to their group to avoid potential cross-contamination.

#### Sample preparation

**Figure 5:**
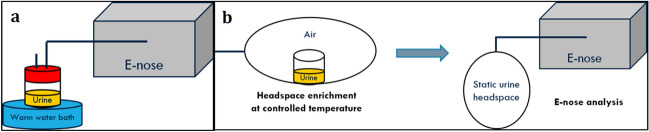
Sample preparation procedure: (**a**) dynamic urine headspace, (**b**) static urine headspace.

The sample preparation procedure was determined after the critical evaluation of eNose sensor responses to the headspaces of the same urine samples obtained in different conditions. This allowed to define a procedure capable of providing stable and reproducible data. Given the ultimate goal of the research of transferring trained dogs’ results to eNose, in the definition on the experimental protocol we tried to limit as much as possible the alterations from the protocol adopted by Taverna et al.^35^. Initially a dynamic headspace was prepared for the eNose analysis (Fig. 5a). The frozen urine sample was thawed, and 30 mL of urine were used for the analysis. The gas flow sent for the analysis was sampled directly from the headspace of a close vessel with a perforated cap, in which the urine was placed and into which odourless air was ducted. The close vessel was put into a water bath at 37 °C, the same temperature of the human body, in order to favour the enrichment of the gaseous headspace and reproduce better the conditions of the analyses with trained dogs.

Those measurements were not reproducible, because dynamic urine headspace evolved during the eNose analysis, thereby resulting in unstable eNose signals. Thus, a static headspace was preferred in order to “freeze” the equilibrium condition reached before the start of the eNose analysis (Fig. 5b). For the creation of static urine headspaces, we used suitable bags in NalophanTM, commonly used for olfactometric analyses, as described in the European Standard EN13725:2003, since this material is characterized by negligible proper odour and chemical inertness. The bags are equipped with a TeflonTM inlet tube, which is used to connect the bag to the electronic nose. Several experimental tests, involving different operating conditions, were considered to define the optimal experimental protocol for enriching the static urine headspace. Initially, the direct heating of the frozen urine sample in the oven was considered and different conditioning temperatures (i.e., 37 °C and 50 °C) and storage times (i.e., 1 h and 2 h) were evaluated. However, the direct heating in oven, carried out at 37 °C and 50 °C, was not adequate for thawing urine samples, because the process did not end within the 2 h. In particular, the enrichment of the urine headspace did not occur always in the same way and eNose signals relevant to the replications of the analysis of the same urine samples different considerably. Consequently, this enriching procedure resulted not reproducible. With the purpose of improving the stability of sensor responses and the reproducibility of the analysis, the defrosting process and the enrichment phases were divided: the urine sample was thawed in a warm water bath, and then, the urine headspace was enriched by heating the liquid sample in the oven at fixed temperature. Different conditioning temperatures (i.e., 23 °C, 37 °C, 50 °C, 60 °C) and storage times (i.e., 1 h, 1.30 h and 2 h) were considered.

#### Electronic nose analysis

For the analysis, the static urine headspace is fluxed into the eNose sensor chamber for 50 min (i.e. during) with a flowrate of 50 mL min^−1^. The interaction between the sensor surface and sample VOCs results in a change of sensor resistance, which is recorded for subsequent elaborations. Then, odourless air is applied to the sensor array with a flowrate of 50 mL min^−1^ for 30 min to restore the reference line (i.e., after). During testing session, the temperature and humidity in the chamber were controlled by means of a climatic chamber to minimize potential interferences on sensor responses toward urine headspaces: analyses were carried out at a temperature of 60 °C and relative humidity of 10%.

### Data processing

The developed procedure for processing eNose data relevant to urine headspace analyses can be described as the sequence of four different stages: signal pre-processing, feature extraction, feature selection, building of classification model. Signal pre-processing involves the removal of baseline shift among analyses carried out over different days by means of Standard Normal Value (SNV) technique^65^, which was applied to resistance curves reordered during the analysis. The second step of the data processing involves the extraction of information relevant for pattern recognition from the sensors response curves. Features, including steady-state and transient responses (Table 5), were extracted from the resistance curve of all the sensors of the array. Features were evaluated in different points of the sensor curve. The feature set involved for further elaboration is a 150-dimesional space. After feature extraction, the most relevant features for classification must be selected. Feature selection is a dimensionality reduction technique that consists in the selection of a subset of features from all the available ones for subsequent use by a learning algorithm. For the specific application, a model based on Boruta algorithm, that provides a measure of the importance of a feature through the measurement of the loss of classification accuracy caused by a random permutation of feature values between objects^66^, was implemented in R. Finally, for classification, a dedicated code, based on randomForest algortithm (i.e., an esemble classifier commonly used to build predictive models for both classification and regression problems^47,67,68^), was implemented in R to build a PCa diagnosis model.

**Table 5:** Features extracted from sensor response curves.

Feature	Description
A	$C= \frac{{R}_{during}}{{R}_{0}}$
B	$\delta = {R}_{during}- {R}_{0}$
C	$\delta = {R}_{after}- {R}_{0}$
D	Minimum value of resistance reached during the measurement (R_min_)
E	$DR= \frac{{R(t}_{3})- {R(t}_{2}) }{{R(t}_{2})-{R(t}_{1})}$
F	${LD=R(t}_{2})- {R(t}_{1})$
G	$y\left[k\right]=\left(1-\alpha \right)y\left[k-1\right]+ \alpha (x\left[k\right]-x\left[k-1\right])$
H	R_0_/R_min_

### Parameters for the evaluation of the diagnostic test

For evaluating the diagnostic capability of the innovative tool, the results of the eNose classification were organized in a confusion matrix, and the classification performance of the innovative diagnostic tool was assessed in terms of accuracy, specificity, and sensitivity. The caret package implemented in Rstudio was used for this evaluation.
